# Prevalence and Risk Factors for Musculoskeletal Disorders in Medical Students

**DOI:** 10.3390/jfmk10040392

**Published:** 2025-10-09

**Authors:** Rogério Rodrigo Ramos, José Maria Pereira de Godoy

**Affiliations:** 1 Department of Anatomy, Santa Fe do Sul University Centre, Santa Fe do Sul 15775-000, SP, Brazil; 2 Postdoctoral Program in Health Sciences, Faculty of Medicine of Sao Jose do Rio Preto (FAMERP), Sao Jose do Rio Preto 15090-000, SP, Brazil; godoyjmp@gmail.com

**Keywords:** anatomy, musculoskeletal pain, academics, ergonomics, occupational health

## Abstract

**Background:** Musculoskeletal disorders are disorders that affect bones, muscles, and joints, significantly impacting quality of life and academic performance. Medical students are particularly susceptible to these conditions due to academic overload, inadequate posture, and overuse of digital devices. **Objectives:** This study aimed at investigating the prevalence of musculoskeletal disorders and associated factors among medical students at a university centre in northwestern São Paulo State. **Materials and Methods:** This cross-sectional, descriptive, and analytical study involved administering a structured questionnaire and conducting clinical tests (Phalen and Finkelstein) to assess musculoskeletal disorders in 164 students. Data were analyzed using the chi-square test with a significance level of 5% (*p*-value < 0.05). **Results:** The results indicate a high incidence of musculoskeletal disorders primarily affecting the upper back, neck and shoulders. In addition, prolonged use of mobile phones and tablets and predominantly typing with the thumbs were found to be associated with an increased risk of De Quervain’s tenosynovitis. A high rate of self-medication was also observed among students, with few participants seeking medical care. These findings highlight the importance of implementing preventive strategies early in the undergraduate curriculum, emphasizing ergonomics and musculoskeletal health awareness. **Conclusions:** This proactive approach can significantly minimize the negative impact on students’ well-being throughout their training and subsequent professional careers. Further studies might explore ergonomic interventions and educational programmes to reduce the incidence of these disorders.

## 1. Introduction

Musculoskeletal disorders (MSDs) encompass a range of conditions affecting bones, joints, muscles, and connective tissues, significantly impacting the functionality of individuals [1]. According to the Global Burden of Diseases [2], these conditions were one of the four primary causes of the absolute increase in disability-adjusted life years between 1990 and 2019. Individuals affected by MSDs may experience a reduced quality of life and difficulties in daily functioning, which can impact academics, work, and social performance. Reduced productivity and inability to work also have direct repercussions for the health system, contributing to absenteeism and a shortage of qualified labour. Despite these significant consequences, MSDs still do not receive the same level of attention or treatment as other occupational and academic diseases [3,4].

Ergonomic and biomechanical factors, such as poor posture, repetitive motions, and excessive force, significantly contribute to the prevalence of MSDs. In the context of occupational health, chronic stress has also been identified as a risk factor, since the high level of academic and work demands, combined with a lack of social and emotional support, can increase an individual’s vulnerability to developing MSDs [5,6]. For health professionals, the pressure to see patients within short time frames can lead to physical and psychological overload, increasing their risk of developing these disorders. Furthermore, Oakman et al. [7] and Anderson and Oakman [8] have described how frequent exposure to emotionally draining situations can aggravate this condition.

Work-related MSDs affect individuals’ functioning and entail significant economic costs for health systems and society. In addition to lost productivity, the need for time off work and prolonged treatment creates a strain on health care services. Although health professionals play a central role in the prevention and management of occupational diseases, studies indicate that they themselves are among the most affected by MSDs. This highlights a gap in the prevention of these conditions among the caregivers themselves [9,10].

However, the effects of MSDs are not limited to professionals already in the job market. Medical students, due to their heavy workload, inadequate postures during long periods of study, and exposure to multiple academic and extracurricular demands, are also susceptible to developing these disorders [11]. This is of particular concern as it may predispose some future doctors to developing MSDs early in their careers, increasing the risk of complications throughout their careers [12].

Studies such as those by Tavares et al. [13] and Torbey et al. [14] suggest that university students experiencing high academic demand are at an increased risk of developing MSDs, either due to a lack of musculoskeletal conditioning or the adoption of inadequate postures during technical and laboratory training. In addition, the academic pressure to meet deadlines and complete assessments can lead to increased stress levels, which are directly associated with the exacerbation of these disorders.

Based on these aspects, it is essential to recognize the importance of ergonomics management and physical training as integral components of academic training. Evidence indicates that physical exercises are effective at reducing pain and preventing MSD-related disability, reinforcing the importance of educational programmes that promote ergonomic awareness and the promotion of regular physical activity. Furthermore, the literature suggests that musculoskeletal injuries may be associated with physical and psychological exhaustion, which is a relevant factor in the development of occupational stress and burnout during medical graduation [15,16,17].

In light of this, the purpose of this study was to investigate the impact of academic activities on the musculoskeletal health of medical science students, thereby contributing to the development of preventive strategies that reduce ergonomic risk and improve quality of life during medical training. Thus, this study aimed to answer the following question: do academic activities and study conditions represent a significant risk for the development of MSDs among medical students? To this end, the study investigated the prevalence and risk factors associated with MSDs among medical students at a university centre in northwestern São Paulo State.

## 2. Materials and Methods

### 2.1. Participants

This cross-sectional, descriptive, and analytical study evaluated qualitative and quantitative variables using standardized questionnaires by Penkala et al. [12] and Morabito et al. [18] adapted by the researchers responsible for the study.

The study was conducted with 164 medical students from the Santa Fé do Sul University Centre, Brazil using a questionnaire consisting of 15 questions aimed at identifying the incidence and possible findings of MSDs in the medical academic community. The questionnaire was applied only once with an estimated completion time of 15–20 min.

### 2.2. Inclusion and Exclusion Criteria

The study participants comprised medical students over the age of 18 who were enrolled in the first through eighth semesters of their course. Those who did not sign the Informed Consent Form (ICF) and those with a previous medical diagnosis of comorbidities affecting the musculoskeletal or neurological system (e.g., rheumatoid arthritis, osteoarthritis, diabetes mellitus, fibromyalgia, herniated disc, osteoarticular fractures, peripheral neuropathies) and who started treatment before entering the course were excluded from this research.

### 2.3. Data Collection Procedures

Data collection took place between August and September 2024, during a regular academic term (excluding assessment weeks and interruptions), to minimize seasonal variations in academic workload and ensure that the results accurately reflect students’ typical routines.

During this period, students from the 1st, 3rd, 5th, 7th and 8th semesters were invited to participate in the research after they had been fully informed about the study objectives. Those who consented received a structured questionnaire containing 15 multiple-choice questions covering the following domains: sociodemographic (age and sex); habits and health, including body mass index (BMI), classified as follows: normal weight (18.5 to 24.9 kg/m^2^), overweight (25.0 to 29.9 kg/m^2^), obesity class I (30.0 to 34.9 kg/m^2^), obesity class II (35.0 to 39.9 kg/m^2^), and obesity class III (≥40 kg/m^2^) [19], strategies adopted to deal with musculoskeletal pain and search for medical assistance; academic factors (course period and weight of backpack carried); MSDs (presence of pain in a body region during academic activities in the previous six months); and ergonomics and occupational health (exercise before theoretical and practical classes, weekly laboratory workload, type of seat used in laboratories, frequency of repetitive motions in the academic routine, finger most used for typing and time of mobile phone/tablet use per day). Furthermore, they were evaluated using the Phalen and Finkelstein clinical tests.

### 2.4. Application of Clinical Tests

**Figure 1:**
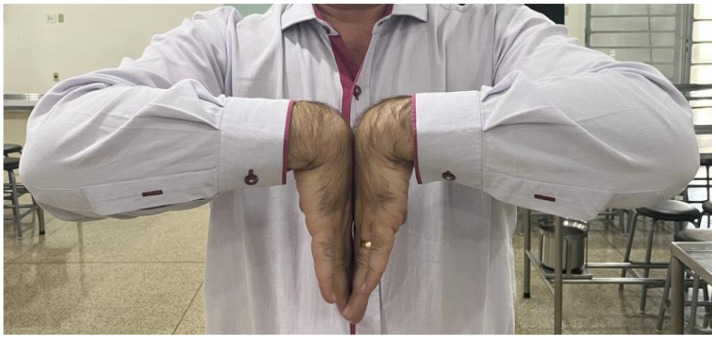
Phalen test.

**Figure 2:**
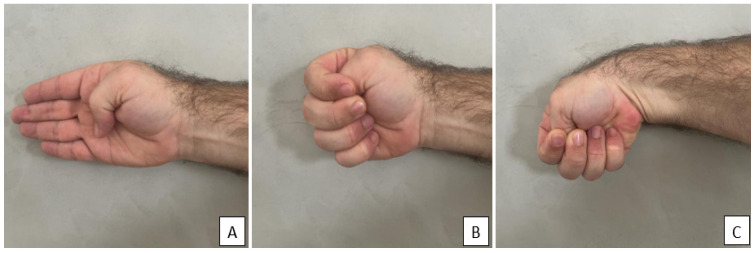
Finkelstein test. (**A**) place their thumb across their palms (**B**) fold their other fingers around their thumbs (**C**) bend their wrist towards their little finger.

Two clinical tests were performed during the application of the questionnaire. For the Phalen test (carpal tunnel syndrome), participants were instructed to fully flex their wrists, bringing the backs of the hands together. They were then asked to hold this position for 60 s (Figure 1). For the Finkelstein test (De Quervain’s tenosynovitis), participants were asked to place their thumb across their palms (Figure 2A), fold their other fingers around their thumbs (Figure 2B) and bend their wrist towards their little finger (ulnar deviation), holding this position for 60 s (Figure 2C). During the clinical tests, some participants reported the existence of pain or discomfort and these data were collected for further analysis.

The Ethical Issues regarding the studies were approved by the Ethics in Research Committee of the Faculty of Medicine of Sao Jose do Rio Preto (FAMERP protocol#CAAE 75143023.4.0000.5415).

### 2.5. Data Analysis

The data were statistically analyzed using frequencies and percentages. Since these are categorical variables, the association analysis was performed using the chi-square test, with a significance level of 5% being considered acceptable (*p*-value < 0.05). The statistical tests were conducted using Minitab 17 software (Minitab Minitab Statistical Software, version 17. State College, PA, USA). 

## 3. Results

Table 1 presents the demographic and academic profiles of the participants. The majority of participants were aged between 18 and 20 years old (42.68%), followed by those aged 21 to 23 years (28.05%). There was a greater proportion of female students (68.29%) in relation to male students (31.71%). Regarding the BMI, most students presented a normal BMI (67.68%), while 23.78% were in the overweight range and 8.54% were obese. Regarding the period of the course, the majority of respondents were in the 1st semester (28.05%), followed by the 3rd semester (27.44%) and 5th semester (21.34%).

Table 2 presents the prevalence of musculoskeletal problems within the previous six months. It reveals that 25.61% of participants did not report any problems during this period, while 39.63% reported three or more musculoskeletal problems. In addition, 45.12% of students reported needing medical assistance or a health professional to treat their musculoskeletal problems. Of these, 18.90% required care for one problem and 12.80% required care for two problems.

Table 3 presents the prevalence of MSDs among students. The upper back was the most commonly affected region, with 48.21% of women and 36.54% of men reporting issues in this area. The neck was the second most affected region (47.32% of women and 38.46% of men), followed by the shoulders (38.39% of women and 34.62% of men). The lower back was another region with considerable prevalence of pain, affecting 32.14% of women and 25% of men. Regarding the extremities, 11.61% of women and 5.77% of men reported pain in the right wrist/hand, while 8.04% of women and 3.85% of men reported pain in the left wrist/hand, suggesting a possible relationship with prolonged use of digital devices. It is also noteworthy that 20.54% of women and 26.92% of men did not present pain during the period analyzed, which indicates that, although the prevalence of MSDs is high, a significant portion of the sample presented no complaints.

Table 4 presents the risk factors associated with musculoskeletal problems. It reveals that the majority of the participants had a weekly workload of up to ten hours (69.51%) and predominantly used stools (72.56%). In addition, a high percentage of students performed repetitive movements (80.49%), and the vast majority used their thumb (87.80%) as their main finger for typing on mobile devices. Most participants (79.88%) reported using mobile phones or tablets for five hours or more daily. A significant proportion of students (37.20%) carried backpacks weighing 2.6 kg or more. The remaining 62.80% of students carried backpacks weighing less than 2.6 kg.

Table 5 presents the association between the number of musculoskeletal problems of wrists/hands reported by students in the previous six months and their preferred finger for mobile device typing. This analysis revealed a significant relationship between these variables. It was observed that the majority of students who use their thumb to type re-ported either no musculoskeletal problems (26.39%) or three or more problems (20.14%), while students who use their index finger had a higher prevalence of two musculoskeletal problems (40.00%).

Table 6 presents the results of the Phalen and Finkelstein tests, used to diagnose carpal tunnel syndrome and De Quervain’s tenosynovitis, respectively. The results provide evidence of the prevalence of these conditions among medical students. It was observed that, among the 164 participants, 23 students (14.02%) had a positive Phalen test for the right hand, while six students (3.66%) had a positive result for the left hand, and 135 students (82.32%) tested negative for both hands. In the Finkelstein test, 42 students (25.61%) had a positive result for the right hand and 16 students (9.76%) for the left hand, while 106 students (64.63%) tested negative for this condition. A significant association was found between the clinical tests and the outcome (positive/negative) observed in each hand. The results specifically revealed a high prevalence of positive cases in the right hand for the Finkelstein test (25.61%) and a high prevalence of negative results for the Phalen test in both hands (85.985 and 96.34%; *p*-value < 0.001).

Table 7 presents the comparative analysis between male and female students in relation to the presence of musculoskeletal pain within the previous six months, showing that pain was slightly more prevalent among women (75.00%) compared to men (73.08%). The initial association was not statistically significant (*p*-value = 0.793).

Table 8 presents the strategies used by students to deal with musculoskeletal pain. Self-medication was the most frequent conduct, reported by 54.88% of students, while 27.44% preferred to wait for the pain to go away of its own accord, 10.98% sought medical care, and 6.70% used alternative treatments. Regarding the adoption of exercises to improve posture before theoretical and practical classes, the majority of students (89.63%) stated that they did not perform any type of exercises, while only 10.37% reported adopting this practice.

Table 9 presents the prevalence of problems encountered by the medical students during their daily activities at the university in the previous six months in relation to the frequency of repetitive movements in their study routine. The results show that the medical students who reported two (21.97%), three (21.97%) or more than three problems (20.45%) also reported repetitive movements in their routine, whereas those who reported no problems (37.50%) reported no repetitive movements.

## 4. Discussion

The findings of this study highlight a high prevalence of MSDs among medical students, influenced by multiple factors, including academic habits, postural overload, excessive use of digital devices, and inadequate pain management strategies. This problem reflects ergonomic and behavioural challenges that can compromise both the well-being of these students and their future professional performance [20]. Borzelli et al. [21] studied the effect of activating two antagonist muscles in a model of the arm for elbow contraction. The authors reported that antagonist muscles potentially contribute to the elbow equilibrium in relation to the muscle activation intensity. In this context, it is possible to assume that antagonist muscles can potentially contribute to musculoskeletal disorders.

The present study analyzed musculoskeletal problems in medical students, given that their demanding routine and frequent lack of rest may impair optimal bodily function and performance of daily and professional activities. Furthermore, medical professionals perform many repetitive hand movements, which cause them to experience problems more frequently than students and professionals from other fields [22,23]. While this study focused on medical students, there is no reason why this type of study could not be conducted with students from other courses or professionals from other fields.

Demographic data reveal a predominantly young student population, with 28.05% of participants aged 21–23 years old. This aligns with the expected age range of students entering this phase of academic life. In addition, the higher proportion of females (68.29%) follows the trend observed in health courses as evidenced in demographic surveys [24]. Regarding the BMI, most students were within the normal weight range, although 23.78% were overweight and 8.54% were obese: this latter category may represent an additional risk factor for MSDs. These findings emphasize the importance of considering not only academic factors, but also the impact of nutritional status on musculoskeletal health. Regarding the course period, the highest participation rate was observed among first-semester students (28.05%), suggesting a greater willingness to contribute to research among newer students compared to those in more advanced semesters.

In the previous six months, 21.95% of students reported three musculoskeletal problems, while 25.61% mentioned no complaints. It is important to highlight that most students reported at least one musculoskeletal problem, evidencing a high prevalence of recurrent episodes of pain, which could affect their quality of life and academic performance. This result indicates that the incidence of MSDs in university students can reach 79.3% as reported by Pacheco et al. [25]. This is particularly high in health courses due to long study hours and prolonged periods spent in uncomfortable positions. This finding is consistent with other investigations, which indicate rates of up to 89.7% [26], demonstrating that this is a widespread problem requiring preventive interventions.

An analysis of the regions most affected by pain reveals a trend toward higher prevalence in the upper back, neck, and shoulders. These findings appear to be in line with previous studies, such as those by Cheung et al. [27] and Kandasamy et al. [28], suggesting that excessive use of digital devices and maintaining static postures for long periods can contribute to musculoskeletal discomfort in these regions. It is possible to assume that inadequate ergonomics in classroom and study environments, combined with few rest breaks, represent an aggravating factor, as has been reported in other studies [12,29]. It should be noted that the causal relationship between these variables was not directly assessed. Future research should therefore specifically investigate the influence of individual ergonomic factors.

Regarding the upper extremities, a proportion of the students reported pain in the wrist and hand. It is noteworthy, however, that Phalen and Finkelstein’s clinical tests identified a higher number of positive cases than self-reported complaints. This finding suggests the presence of subclinical musculoskeletal alterations among the participants, a condition characterized by detectable clinical signs despite the absence of self-reported pain [30]. This hypothesis reinforces the importance of early assessments for screening for MSDs, as recommended by the literature [1,31]. Despite this finding, it is critical to acknowledge that the association between digital device use and wrist/hand pain was not directly analyzed concerning the duration of use between the symptomatic and asymptomatic groups, which represents a limitation and a key suggestion for subsequent research.

The findings highlight multiple factors associated with MSDs, with repetitive motions being reported as a main factor by 80.49% of the students. This pattern can increase biomechanical overload and favour the development of carpal tunnel syndrome and De Quervain’s tenosynovitis [32]. In addition, the use of stools by 72.56% of the students can aggravate musculoskeletal discomfort, as seats without adequate support can lead to postural misalignments and muscle fatigue [33].

Another relevant finding is that 87.80% of the students use their thumb to type on mobile phones and tablets, with 79.88% reporting a daily use of more than five hours. These data suggest that prolonged and repetitive use of these technologies may be related to the development of MSDs [34]. Overloading of the extensor and flexor tendons of the thumb significantly increases the risk of tenosynovitis and other repetitive strain injuries [35]. This relationship is further evidenced by positive clinical test results: 14.02% for Phalen’s test (right hand) and 25.61% for Finkelstein’s test (*p*-value < 0.001). This suggests that excessive use of mobile devices directly impacts the musculoskeletal health of students [36,37].

Complementing this analysis, the study provided a detailed evaluation of the association between the predominant finger used for typing and the number of musculoskeletal problems over a six-month period. The results demonstrated a statistically significant relationship (*p*-value = 0.012), suggesting that a heavy reliance on the thumb may be linked to elevated musculoskeletal complaints. The analysis of the six-month period was relevant to capture both recent and persistent complaints, corroborating the direct influence of the typing pattern on the development of work-related MSDs [38]. These findings underscore the critical importance of implementing ergonomic interventions focused on the conscious use of digital devices within academic routines to mitigate tendon and joint overload.

The way students deal with musculoskeletal pain is a cause for concern. It was observed that most participants (54.88%) chose not to seek medical assistance or a health professional, instead resorting to self-medication as their main strategy for pain control. Meanwhile, only 10.98% sought medical care. This behaviour suggests a tendency to neglect symptoms, favouring the progression of MSDs. Studies such as those by Martinez et al. [39], Tognoli et al. [40] and Lima et al. [41] indicate that self-medication is common among university students due to the high academic workload and difficulty accessing health services.

Furthermore, a gender-based analysis revealed that the overall prevalence of musculoskeletal pain in the previous six months was statistically similar (*p*-value = 0.793) between women (75.00%) and men (73.08%). This finding suggests that, despite equivalent overall pain perception between genders, women may be experiencing multiple episodes of pain of sufficient severity to necessitate professional medical attention within a short time frame. Hormonal factors, lower muscle mass and biomechanical differences may justify this predisposition [42,43].

The analysis between performing repetitive movements in academic routines and a greater number of musculoskeletal problems reveals a statistically significant association (*p*-value = 0.035). The data show that the majority of students who did not engage in repetitive movements reported no problems (37.5%). In sharp contrast, most of those who reported more than one problem (two, three, or more than three) belonged to the group that performed repetitive movements. This result indicates that the repetition of movements, common in activities such as typing and using digital devices, can be considered a relevant risk factor for the development of musculoskeletal disorders among students [44]. It is believed that the absence of breaks and the lack of different movements during long study periods contribute significantly to musculoskeletal overload. Thus, these data reinforce the need for ergonomic and educational interventions that promote awareness of the effect of repetitive movements, the importance of active breaks, and task switching in the academic environment.

In the light of these findings, it is essential to reflect on the academic and professional implications of MSDs. The high incidence of musculoskeletal symptoms not only impairs the performance of students during undergraduate studies, but can also evolve into chronic conditions, impacting their future ability to work [25,45]. Lack of awareness about ergonomics, combined with neglect of musculoskeletal health care, can result in progressive conditions that require more complex therapeutic interventions [46,47].

Thus, the results of this study reinforce the need for preventive and educational actions, in addition to the implementation of ergonomic strategies that can minimize the impact of MSDs. The progressive introduction of chairs with adjustable lumbar support, along with guidance on proper seat adjustment, is recommended to reduce postural overload and improve furniture ergonomics. It is also suggested that active breaks be incorporated during long study periods, together with exercise programmes focused on strengthening core muscles and stretching, in order to increase muscular endurance and reduce fatigue. Finally, it is important to implement educational programmes on ergonomic practices for technology use, including guidance on varying finger positions when typing, keeping wrists in a neutral position, limiting continuous use of mobile devices, and performing specific hand- and wrist-stretching exercises.

Implementing these measures into the academic curriculum, represents a fundamental step in mitigating the impact of MSDs on students’ well-being and their future clinical practice.

Therefore, it is essential to invest in raising awareness of musculoskeletal health among undergraduate students to ensure that future health professionals are able to maintain their quality of life and clinical performance throughout their careers.

Although this study provides valuable insights into the risk factors for MSDs in medical students, it is crucial to consider its methodological limitations for an adequate interpretation of the findings.

### Limitations of the Study

The exclusion of participants with prior comorbidities (arthritis, diabetes, traumatic injuries, fibromyalgia, etc.) was a methodological choice intended to control for potential confounding bias, as these conditions can mimic or worsen MSDs. However, this reduces the generalizability of the results to medical students with these conditions and fails to capture undiagnosed cases (e.g., subclinical fibromyalgia), which could influence reported pain perception. Future studies should therefore include active screening for comorbidities.

Furthermore, as the study was conducted at a single educational institution in northwestern São Paulo State, the results only reflect the reality of this specific context, limiting the extrapolation of the findings to different regional or national contexts. Multicentre studies involving universities in different regions are needed to confirm the prevalence of the MSDs and risk factors identified here.

Moreover, the Phalen and Finkelstein tests were chosen as rapid screening tools, considering their applicability in academic settings. However, we recognize that definitive diagnoses of carpal tunnel syndrome and De Quervain’s tenosynovitis require additional tests (e.g., electroneuromyography, ultrasound), a factor that may have underestimated the true prevalence of these conditions.

## 5. Conclusions

This study identified a significant incidence of MSDs in future health professionals, influenced by factors such as inadequate posture, prolonged exposure to mobile phones and tablets and ineffective management of musculoskeletal pain. The most affected regions were the upper back, neck and shoulders, reflecting the overload associated with the academic environment. In addition, the continuous use of these mobile devices, combined with the predominance of thumb typing, was shown to increase the risk of De Quervain’s tenosynovitis.

These findings meet the research objective of demonstrating that both ergonomic factors and academic habits influence the occurrence of MSDs. The high rate of self-medication among medical students, combined with the low rate of seeking professional medical care, underscores the urgent need for preventive and educational interventions to mitigate the impact of these disorders throughout their training.

Therefore, this study highlights the crucial need for preventive strategies that emphasize ergonomics and musculoskeletal health awareness. These should be implemented early in the undergraduate curriculum, encouraging strategies such as postural adjustments, regular breaks and the promotion of physical activity. For future research, we suggest expanding the sample to other institutions and carrying out longitudinal studies, in addition to evaluating the effectiveness of ergonomic interventions and educational programmes in reducing the incidence of MSDs.

## Figures and Tables

**Table 1:** Demographic and academic profile of the participants.

Variable		*n*	%
Age range	18–20 years	70	42.68
	21–23 years	46	28.05
	24–26 years	20	12.20
	27–30 years	11	6.71
	More than 30 years	17	10.37
Sex	Female	112	68.29
	Male	52	31.71
BMI	Normal	111	67.68
	Overweight	39	23.78
	Obese Grade I	13	7.93
	Obese Grade II	1	0.61
Stage in course	1st semester	46	28.05
	3rd semester	45	27.44
	5th semester	35	21.34
	6th semester	1	0.61
	7th semester	16	9.76
	8th semester	21	12.80

BMI: body mass index.

**Table 2:** Prevalence of musculoskeletal problems in the previous six months.

Problem		*n*	%
In the previous six months, how many problems have you encountered during your daily activities at the University?	None	42	25.61
	One	25	15.24
	Two	32	19.51
	Three	36	21.95
	More than three	29	17.68
In the previous six months, how many problems have you had that required medical assistance or a health professional?	None	90	54.88
	One	31	18.90
	Two	21	12.80
	Three	11	6.71
	More than three	11	6.71

**Table 3:** Prevalence of musculoskeletal disorders in the previous six months by body region.

Affected Region	Female *n* (%)	Male *n* (%)
Upper back	54 (48.21)	19 (36.54)
Neck	53 (47.32)	20 (38.46)
Shoulders	43 (38.39)	18 (34.62)
Lower back	36 (32.14)	13 (25)
Did not experience any pain	23 (20.54)	14 (26.92)
Right knee	18 (16.07)	4 (7.69)
Right wrist/hand	13 (11.61)	3 (5.77)
Left knee	10 (8.93)	5 (9.62)
Left wrist/hand	9 (8.04)	2 (3.85)
Right foot/ankle	8 (7.14)	2 (3.85)
Left foot/ankle	7 (6.25)	1 (1.92)
Hips/thighs	5 (4.46)	3 (5.77)

**Table 4:** Risk factors related to musculoskeletal problems.

Risk Factor	Category	*n*	%
Weekly workload	Up to 10 h	114	69.51
	11–20 h	18	10.98
	More than 20 h	32	19.51
Main seat type	Stool	119	72.56
	Adjustable chair	45	27.44
Performed repetitive body movements	Yes	132	80.49
	No	32	19.51
Finger used when using the digital device	Thumb	144	87.80
	Index finger	20	12.20
Daily mobile phone/tablet usage	5 h or more	131	79.88
	Up to 4 h	33	20.12
Backpack weight	2.6 kg or more	61	37.20
	Up to 2.5 kg	103	62.80

**Table 5:** Association between musculoskeletal problems and the finger used for typing on digital devices.

How Many Problems Have You Encountered During Your Daily Activities at the University in the Last Six Months?	Finger Used for Typing	
	Index Finger	Thumb
None	4 (20.00%)	38 (26.39%)
One	2 (10.00%)	23 (15.97%)
Two	8 (40.00%)	24 (16.67%)
Three	6 (30.00%)	30 (20.83%)
More than three	0 (0.00%)	29 (20.14%)
*p*-value ^1^ = 0.012		

^1^ The Chi-square test indicated statistical significance (*p*-value < 0.05).

**Table 6:** Clinical Tests for Diagnosis of carpal tunnel syndrome and De Quervain’s tenosynovitis.

Category	Phalen Test		Finkelstein Test	
	*n*	%	*n*	%
Right positive	23	14.02	42	25.61
Left positive	6	3.66	16	9.76
Negative	135	82.32	106	64.63

**Table 7:** Comparison of pain prevalence between genders.

Variable	Category	Male		Female	
		*n*	%	*n*	%
Pain in the last six months	Yes	38	73.08	84	75.00
	No	14	26.92	28	25.00
*p*-value = 0.793					

**Table 8:** Strategies adopted by students to deal with musculoskeletal pain.

Risk Factor	Category	*n*	%
What do you do when you feel some kind of pain in your body?	Self-medicate	90	54.88
	Wait for the pain to pass alone	45	27.44
	Find a doctor	18	10.98
	Uses alternative treatments	11	6.70
Do you do exercises to improve your posture before theoretical and/or practical classes?	No	147	89.63
	Yes	17	10.37

**Table 9:** Association between the prevalence of problems of the medical students during their daily activities and the frequency of repetitive movements during their study routine.

Variable	Category	Repetitive Movements			
		No		Yes	
		*n*	%	*n*	%
How many problems have you encountered during your daily activities at the University in the last six months?	None	12	37.50	30	22.73
	One	8	25.00	17	12.88
	Two	3	9.38	29	21.97
	Three	7	21.88	29	21.97
	More than 3	2	6.25	27	20.45
*p*-value = 0.035					

## Data Availability

Data are available upon reasonable request.
